# Clinical effect of digitalized designed and 3D-printed repositioning splints in the treatment of anterior displacement of temporomandibular joint disc

**DOI:** 10.1186/s12891-024-07477-z

**Published:** 2024-05-03

**Authors:** Xiao Jin, Wang Chi

**Affiliations:** Department of Oral and Maxillofacial Surgery, School & Hospital of Stomatology, Wenzhou Medical University, Wenzhou, Zhejiang 325000 China

**Keywords:** Digitalized design, 3D printed, Anterior repositioning splint, Anterior disc displacement of the temporomandibular joint

## Abstract

**Objective:**

To compare the treatment effectiveness of digitized and 3D-printed repositioning splints with that of conventional repositioning splints in the treatment of anterior displacement of the temporomandibular joint disc.

**Methods:**

This retrospective study included 96 patients with disc displacement of the anterior temporomandibular joint. They were treated with either digitally designed and 3D-printed repositioning splints or traditional splints and followed up for at least six months. Changes in signs and symptoms such as pain and mouth opening before and after treatment were recorded to evaluate treatment outcomes.

**Results:**

During the first month of treatment, both the digitally designed and 3D-printed repositioning splint groups (Group B) and the traditional repositioning splint group (Group A) showed significant increases in mouth opening, with increases of 4.93 ± 3.06 mm and 4.07 ± 4.69 mm, respectively, and there was no significant difference between the two groups. Both groups had a significant reduction in visual analog scale (VAS) pain scores, with Group B showing a greater reduction of 1.946 ± 1.113 compared to 1.488 ± 0.978 in Group A (*P* < 0.05). By the sixth month, Group B’s mouth opening further improved to 38.65 ± 3.22 mm (*P* < 0.05), while Group A’s mouth opening did not significantly improve. Regarding pain, Group A’s VAS score decreased by 0.463 ± 0.778 after one month, and Group B’s score decreased by 0.455 ± 0.715; both groups showed significant reductions, but there was no significant difference between the two groups.

**Conclusion:**

Compared with traditional repositioning splints, digitally designed and 3D-printed repositioning splints are more effective at reducing patient pain and improving mouth opening. 3D-printed repositioning splints are an effective treatment method for temporomandibular joint disc displacement and have significant potential for widespread clinical application.

## Background

Temporomandibular disorders include a range of conditions affecting the oral and maxillofacial systems. Studies indicate a prevalence of 10 to 28% [1, 2]. The etiology of temporomandibular disorder is complex. Previous studies have shown that it is multifactorial and involves occlusal [3], psychological [4], traumatic [5], habitual [6], and general health [7–9] factors. Other studies have suggested that age is associated with TMJ disorders [10]. Further studies have shown that TMD may be related to cervical spine disorders and mobility [11–13].

These disorders can lead to symptoms such as pain, restricted mouth opening, and joint clicking and are therefore among the main causes of orofacial pain. Among these defects, anterior displacement of the disc is the most common [14, 15]. Displacement of the TMJ disc can lead to serious problems such as condylar resorption and craniofacial abnormalities in adolescents [16–18]. The treatment of temporomandibular joint disc displacement is therefore becoming increasingly important. Patient education, exercise, physical therapy, occlusal splints and surgery have been performed [19, 20], among which splint therapy is one of the most common clinical approaches for treating temporomandibular joint disorders [21], and repositioning splints is considered an effective treatment for anterior disc displacement [22–24]. In the traditional fabrication of repositioning splints, the process includes determining the jaw position, making impressions, establishing occlusal relationships, transferring the clinically determined jaw position to the articulator, and finally delivering the splint to the patient after fabrication and fitting in the laboratory. This entire process inevitably requires multiple transfers of models and occlusion relationships, which introduces a certain degree of error and has a negative impact on the accuracy of the splint. With the development of digitally assisted design and 3D printing technology, several scientists have attempted to utilize digital technology and 3D printing for splint manufacturing [24]. However, the jaw position and occlusion relationships still need to be digitized after the impression is taken and the model is scanned [25], and there is insufficient research on their effectiveness. Therefore, we hypothesized that the use of a repositioning splint via digital scanning and 3D printing could achieve better treatment results. In this study, we aimed to utilize digital techniques for data collection, including jaw position recordings and a splint design, for patients with anterior disc displacement. We used 3D printing technology to produce repositioning splints through digitalization. These splints were then compared to conventional repositioning splints for further analysis.

## Materials and methods

### Study subjects and groups

This study received approval from the Ethics Committee of the Affiliated Stomatology Hospital, Wenzhou Medical University (Ethics number: WYKQ2021004). The study collected data from patients who visited the Temporomandibular Joint Specialist Clinic at the Affiliated Stomatology Hospital of Wenzhou Medical University from January 2021 to September 2022. In these patients, magnetic resonance imaging confirmed displacement of the temporomandibular joint disc with corresponding clinical symptoms. The inclusion criteria were as follows: (1) diagnosis of disc displacement confirmed by magnetic resonance imaging; (2) no history of occlusal treatment or temporomandibular joint surgery; and (3) a follow-up duration exceeding six months. The exclusion criteria were as follows: (1) patients who were uncooperative with treatment or had poor compliance; (2) patients with other painful oral diseases, such as pulpitis or periapical periodontitis; (3) patients in the acute phase of any disease; (4) patients with severe periodontal disease; and (5) patients with incomplete medical records or less than six months of follow-up.

The patients were divided into two groups based on the method of occlusal splint preparation: the traditional repositioning splint group (Group A) and the digitalized personalized splint group (Group B). At the first visit, detailed medical histories and pretreatment signs (T0) were recorded, including sex, age, disease progression, mouth opening (Z0), and visual analog scale (VAS) score for pain (VAS0). The VAS score was measured with a visual analog scale by the same doctor, and a reduction in the VAS score was considered effective. The mouth opening was measured as follows: (1) The patient sat up straight with the head and torso in a straight line. (2) The patient opened the mouth as much as possible. (3) The mouth-opening meter was placed parallel to the ground on the patient’s upper and lower dental arches.

### Treatment process

#### Digitalized design and 3D-printed groups

A TRIOS dental scanner (3Shape, Denmark) was used to capture the upper and lower dental arches and the occlusal relationship. The data were imported into the virtual articulator of the exocad design software V3.0 (Amann Girrbach, Germany), and an auxiliary instrument, a balancer, was designed to determine the patient’s jaw position. The balancer was fabricated by a Heygear 3D printer (UltraCraft A3D, Heygear, China) using high-precision dental mold resin (Model HP UV 2.0, Heygear, China).

The patient wore the balancer to achieve a stable jaw position through a process called muscle deprogramming [26]. Bite registration silicone rubber (O-bite, DMG, Germany) was used to record the patient’s current upper and lower jaw relationships. These data were imported into Exocad design software V3.0 (Amann Girrbach, Germany) to design the mandibular splint. The splint was semianatomically designed. Uniform contact between the upper posterior teeth and the splint in the target jaw position with a cusp-fossa locking relationship was required. At the same time, the cusp sliding on the splint was not disturbed. There was no contact between the upper anterior teeth and the occlusal splint, and the design also ensured protection of the anterior teeth without premature contact or interference during protrusive and lateral movements.

The occlusal splint was prepared with temporary crown bridge resin (Yamahachi, Japan) by using a five-axis engraving machine (Wieland, Germany), and the thickness of the splint was 2–3 mm. Once completed, the patient wore the splint for the initial fitting and adjustment, and the time spent in adjustment on the chair was recorded. Patients wore the splint 24 h/day. Follow-up visits were scheduled; one week after wearing the splint, the patient returned for a follow-up visit for adjustment. Adjustments were made if necessary, with the objectives described above. All patients underwent similar treatment procedures.

A follow-up visit was conducted after one month (T1) to make adjustments and evaluate changes in clinical symptoms, including mouth opening (Z1) and visual analog scale pain (VAS 1) scores. Subsequent monthly follow-up visits included splint adjustments and assessments of changes in clinical symptoms. The signs and symptoms, including mouth opening (Z2) and pain VAS score (VAS2), were collected at the sixth month (T2).

#### Traditional occlusal splint group

For the impressions, polyvinyl siloxane (Enormous, China) was used and poured into a mold to create models of the upper and lower dental arches. The impressions were rinsed with running water, blow-dried, immersed in 0.5% sodium hypochlorite solution for 10 min, rinsed again for 15 s, and blow-dried again. The models were made of die stone (SSS, Japan). The target position of the jaw was determined based on the disappearance of anterior mandibular displacement and the use of a click during protrusion. The patient’s occlusal relationship was recorded using polyvinyl siloxane (O-bite, Germany) for bite registration.

The upper and lower arch models were transferred along with the occlusion relationship to a mechanical articulator (Ivoclar, Switzerland). The bite splint for repositioning was designed using the same principles as those used for the 3D-printed splint group.

Dental technicians used self-curing resin (Nissin, Japan) to prepare the repositioning occlusal splint according to the above design and then polished and finished the splint. A mandibular splint was produced, and the thickness of the splint was 2–3 mm. The patient wore the occlusal splint for the initial fitting and adjustment. Patients wore the splint 24 h/day. Follow-up examinations were planned. One week after wearing the splint, the patient returned for a follow-up examination for adjustment. Adjustments were made if necessary, with the objectives described above. A follow-up visit was performed after one month (T1) to make adjustments and assess changes in clinical symptoms, including mouth opening (Z1) and visual analog scale (VAS1) pain. Subsequent monthly follow-up visits included splint adjustments and assessments of changes in clinical symptoms. The signs and symptoms, including mouth opening (Z2) and pain VAS (VAS2) scores, were collected at the sixth month (T2). All patients underwent similar treatment procedures.

### Statistical analysis

The data were statistically analyzed using IBM SPSS Statistics for Windows, version 21.0 (IBMCorp, Armonk, N.Y. USA). For numerical data, including age, mouth opening, duration, and changes in pain scores, paired t tests were used for comparisons before and after treatment within the same group. A *P* < 0.05 indicated a statistically significant difference. Chi-square tests were used to analyze sex distributions and treatment effectiveness. A significance level of *P* < 0.05 was considered to indicate statistical significance.

## Results

### Baseline information

This study included a total of 96 eligible patients, 41 of whom were in the conventional occlusal splint group (Group A), including 32 females and 9 males, with a mean age of 25.95 ± 5.21 years. The 3D-printed splint group (Group B) included 55 people—40 females and 15 males—with an average age of 26.98 ± 3.85 years. There were no significant differences in age, sex distribution, pretreatment mouth opening, or VAS score between the two groups (Table 1).

**Table 1:** Baseline information

Group		Age (y)	mouth opening at T0 (mm)	VAS at T0
Group A	41 (female 32, male 9)	25.95 ± 5.21	34.17 ± 5.33	3.707 ± 1.188
Group B	55 (female 40, male 15))	26.98 ± 3.85	33.02 ± 4.10	3.400 ± 1.226

Before treatment, the baseline pain levels (VAS 0) in the two groups were 3.707 ± 1.188 and 3.400 ± 1.226, respectively, with no significant difference (*P* > 0.05). Considering the effectiveness of pain relief after treatment, the effectiveness rate in Group A was 82.9% (34/41) after one month, while in Group B, it was 87.27% (48/55). After six months, the effectiveness was 85.4% in Group A and 94.5% in Group B. The chi-square test showed no significant difference in effectiveness between the two groups. In the longitudinal comparison, both Group A and Group B showed a continuous decrease in the pain index, and this decrease was significant at each treatment time point (*P* < 0.05) (Table 2). According to the cross-sectional comparison, the reduction in the VAS score (VAS 1) after one month was significantly greater in Group B than in Group A (*P* < 0.05). However, at the sixth month, there was no significant difference in the reduction in VAS score (VAS 2) between the two groups compared to the VAS score of 0 (Table 3).

**Table 2:** VAS changes in different treatment groups

Group	VAS 0	VAS 1	Effective rate at T1	VAS 2	Effective rate at T2
Group A	3.707 ± 1.188	2.220 ± 0.852*	82.9%	1.561 ± 0.808*	85.4%
Group B	3.400 ± 1.226	1.455 ± 0.997#*	87.3%	1.000 ± 0.839#*	94.5%

# Significant difference between groups (*P* < 0.05). *Significant difference within the group compared to the previous VAS score (*P* < 0.05).

**Table 3:** Comparison of VAS score decrease between different treatment groups

Group	VAS 1-VAS0	VAS 2-VAS1	VAS 2-VAS0
Group A	-1.488 ± 0.978	-0.463 ± 0.778	-1.951 ± 1.161
Group B	-1.946 ± 1.113#	-0.455 ± 0.715	-2.400 ± 1.099

#There is a significant difference in comparison between groups during the same time period (*P* < 0.05)

The maximum mouth opening (Z0) before treatment was 34.17 ± 5.33 mm and 33.02 ± 4.10 mm at baseline in both groups, respectively, with no significant difference (*P* > 0.05). In a longitudinal comparison, both Group A and Group B showed continuous improvement in mouth opening. At the first-month follow-up, the maximum mouth opening was 38.24 ± 3.38 mm and 37.95 ± 3.59 mm for the two groups, respectively, both of which were significantly different. However, at the six-month follow-up (compared to that at one month), there was no significant change in maximum mouth opening in the conventional treatment group, while there was a significant improvement in the 3D-printed group during this treatment period (*P* < 0.05) (Table 4). However, in a cross-sectional comparison, there was no significant difference in the degree of improvement in maximum mouth opening between groups at any time point (Table 5).

**Table 4:** Mouth opening of different treatment groups

Group	Z0(mm)	Z1(mm)	Z2(mm)
Group A	34.17 ± 5.33	38.24 ± 3.38*	38.61 ± 3.26
Group B	33.02 ± 4.10	37.95 ± 3.59*	38.65 ± 3.22*

*There is a significant difference in mouth opening within the group compared to the previous time(*P* < 0.05)

**Table 5:** Improvement of mouth opening in different treatment groups

Group	Z1-Z0(mm)	Z2-Z1(mm)	Z2-Z0(mm)
Group A	4.07 ± 4.69	0.37 ± 1.57	4.44 ± 4.76
Group B	4.93 ± 3.06	0.71 ± 1.33	5.63 ± 2.91

## Discussion

Temporomandibular joint anterior disc displacement is a common type of temporomandibular joint disorder. In the 1970s, Farrar [27] first proposed the use of repositioning splints to treat anterior disc displacement of the temporomandibular joint. It is believed that the basic principle is to improve the position of the condyle in the articular fossa, thereby achieving a normal disc-condyle relationship and improving mandibular function, resulting in therapeutic effects.

Our study showed that both traditional repositioning splints and 3D-printed splints were effective at reducing patients’ pain in the first month of wear, and this effect was maintained until the sixth month of follow-up. This relief was more pronounced in the 3D-printed group. In addition, both groups showed significant improvement in mouth opening in the first month of treatment. However, the 3D-printed splint group showed slight sustained improvement after six months, while the traditional splint group showed no further improvement. The cause of these results could be related to the origin of symptoms in the displacement of the anterior disc of the temporomandibular joint. Pain in temporomandibular joint diseases mainly arises from muscle pain caused by muscle dysfunction and intra-articular pain caused by joint structural disorders [28]. Although some researchers believe that disc displacement has nothing to do with clinical symptoms and dentofacial deformities [29], further studies have shown that changes in disc position increase the risk of joint degenerative changes and joint swelling, resulting in clinical manifestations such as pain and limited mouth opening leads [30]. In addition, research by Dias [31] revealed a significant association between disc displacement and osteoarthritis, and osteoarthritis was significantly associated with bone marrow edema and pain [32]. Repositioning splints can relieve joint swelling by improving the disc‒condyle relationship, thereby improving clinical symptoms. In addition, splint therapy can effectively relieve muscle-related pain. According to several electromyographic studies, in temporomandibular joint diseases, the lateral pterygoid muscle must contract abnormally to resist the elevator muscle, which may lead to excessive activation and coordination of the masticatory neuromuscular tissue, resulting in orofacial pain. After splint therapy, muscle activity decreases, the load on the temporomandibular joint decreases, and the maximum contraction force decreases, thereby relieving pain [33]. Therefore, both stabilizing splints and repositioning splints have significant therapeutic effects on pain caused by temporomandibular joint disorders [20].

Both traditional repositioning splints and 3D-printed splints can improve patients’ clinical symptoms to a certain extent, as manifested by pain relief and improved mouth opening. The effective rates in the first month were 82.9% and 87.3%, respectively, similar to those in Lei’s (78.1%) and Liu’s (83.78%) studies [34, 35] but higher than those in Schmitter’s study (50%) [36]. The variety of causes of TMJ disc disorders, such as different age distributions and psychological statuses [37], may have led to significant differences in the effectiveness of treatment among different studies. In terms of clinical effectiveness, the 3D-printed splint group still outperformed the traditional repositioning splint group in terms of pain relief. By analyzing the differences between these two treatment plans, we found that traditional repositioning splints rely on manual determination of the target occlusion, which has the disadvantage that the disc‒condyle relationship cannot be directly assessed. Once there is a significant deviation between the actual occlusion and the expected occlusion, the treatment effect will be significantly affected. Additionally, traditional repositioning splints rely on the clinical experience and finesse of the doctor and technician. The accumulated errors in each step of the production process may have a significant impact on the clinical outcome. The cumulative effect of these factors ultimately affects the treatment effect. In contrast, 3D-printed splints combine occlusion with digital image data via a computer, achieving more ideal occlusion. Compared to the occlusion of traditional repositioning splints, this method determines the occlusion by directly observing the position relationship between the condyle and the joint fossa. The determined occlusion can be transferred directly to the digital design and production of repositioning splints, thereby reducing errors in subsequent processes. The use of 3D printing technology also contributes to the accuracy of splint manufacturing, resulting in better treatment effects. In addition, the resin material used in the repositioning splint of the 3D printing group was not the same as that used in the traditional group, which may also have a certain impact on the treatment effect [38–40], and the performance of these two resins when used as jaw pad materials requires further investigation.

Another important difference between 3D-printed splints and traditional splints is the deprogramming of the muscles. In this procedure, a stabilizer is first created to release the patient’s posterior teeth from occlusal contact. Standardized mandibular movements are then used to fatigue the masticatory muscles and achieve stable occlusion, thereby reducing the influence of subjective factors and avoiding repeated splint adjustments during subsequent clinical follow-up. This technology reduces, to a certain extent, the differences caused by the operator’s personal judgment in 3D-printed splint therapy. In contrast, occlusion in traditional splint fabrication depends solely on the dentist’s clinical experience. Using digital technology allows for more accurate constructions and precise balancing of occlusal relationships [41]. This could be one of the reasons why the 3D-printed splint group still showed an improvement in maximum mouth opening after six months, while the traditional splint group did not.

Our study showed that 3D-printed repositioning splints are more effective than traditional repositioning splints in improving clinical symptoms caused by displacement of the anterior disc of the TMJ [10]. In addition, research suggests that the use of digital technology can simplify the splint manufacturing process and reduce the working time of technicians and doctors, which is consistent with our clinical observations [25]. Therefore, digitized and 3D-printed repositioning splints have good clinical value.

This study has several limitations. First, due to the short follow-up period of this study, it is not yet possible to provide a clear answer about the long-term effect, and further observations and analyses are necessary. More influencing factors should also be considered. Second, the evaluation of efficacy in this study is limited to the relief of clinical symptoms, and there is a lack of measurement and analysis of imaging data such as MRI findings. The combination of clinical symptoms and imaging data will be a major part of our further investigation.

## Conclusion

Due to the advantages of digital technology in occlusion selection and the precision offered by 3D printing, 3D-printed repositioning splints are more effective than traditional repositioning splints in relieving patient pain and improving mouth opening. They are effective treatments for temporomandibular disc dislocation and have significant value for wide clinical application.

## Data Availability

The datasets used and/or analyzed during the current study are available from the corresponding author upon reasonable request.

## References

[1] Poveda R, Bagan JV, Díaz JM (2007). Review of temporomandibular joint pathology. Part I: classification, epidemiology and risk factors. Med Oral Patol Oral Cir Bucal.

[2] Tomas X, Pomes J, Berenguer J (2006). MR imaging of temporomandibular joint dysfunction: a pictorial review. Radiographics.

[3] Thomas DC, Singer SR, Markman S. Temporomandibular Disorders and Dental Occlusion: What Do We Know so Far? Dent Clin North Am. 2023;67(2):299–308. 10.1016/j.cden.2022.11.002. Epub 2023 Feb 1. PMID: 36965932. 10.1016/j.cden.2022.11.002 36965932

[4] Simoen L, Van den Berghe L, Jacquet W (2020). Depression and anxiety levels in patients with temporomandibular disorders: comparison with the general population. Clin Oral Investig.

[5] Häggman-Henrikson B, List T, Westergren HT et al. Temporomandibular disorder pain after whiplash trauma: a systematic review. J Orofac Pain. 2013 Summer;27(3):217 – 26. 10.11607/jop.1027. PMID: 23882454. 10.11607/jop.1027 23882454

[6] Topaloglu-Ak A (2022). Can sleeping habits be associated with sleep bruxism, temporomandibular disorders and dental caries among children?. Dent Med Probl.

[7] Minervini G, Marrapodi MM, La Verde M (2024). Pregnancy related factors and temporomandibular disorders evaluated through the diagnostic criteria for temporomandibular disorders (DC/TMD) axis II: a cross sectional study. BMC Oral Health.

[8] Bueno CH, Pereira DD, Pattussi MP (2018). Gender differences in temporomandibular disorders in adult populational studies: a systematic review and meta-analysis. J Oral Rehabil.

[9] Seweryn P et al. Relationship between pain severity, satisfaction with life and the quality of sleep in Polish adults with temporomandibular disorders [published online as ahead of print on October 24, 2023]. Dent Med Probl. 10.17219/dmp/171894. 10.17219/dmp/171894 37873974

[10] Poluha RL, Canales GT, Costa YM et al. Temporomandibular joint disc displacement with reduction: a review of mechanisms and clinical presentation. J Appl Oral Sci. 2019;27:e20180433. 10.1590/1678-7757-2018-0433. Erratum in: J Appl Oral Sci. 2019;27:e2019er001. PMID: 30810641; PMCID: PMC6382319. 10.1590/1678-7757-2018-0433 PMC6382319 30810641

[11] Wiesinger B, Malker H, Englund E (2009). Does a dose-response relation exist between spinal pain and temporomandibular disorders?. BMC Musculoskelet Disord.

[12] Kim D, Ko SG, Lee EK (2019). The relationship between spinal pain and temporomandibular joint disorders in Korea: a nationwide propensity score-matched study. BMC Musculoskelet Disord.

[13] Boening K (2015). Temporomandibular disorders and oral parafunctions: mechanism, diagnostics, and therapy. Biomed Res Int.

[14] Murphy MK, MacBarb RF, Wong ME, Athanasiou KA. Temporomandibular disorders: a review of etiology, clinical management, and tissue engineering strategies. Int J Oral Maxillofac Implants. 2013 Nov-Dec;28(6):e393–414. 10.11607/jomi.te20. PMID: 24278954; PMCID: PMC4349514. 10.11607/jomi.te20 PMC4349514 24278954

[15] Manfredini D, Guarda-Nardini L, Winocur E (2011). Research diagnostic criteria for temporomandibular disorders: a systematic review of axis I epidemiologic finding. Oral Surg Oral Med Oral Pathol Oral Radiol Endod.

[16] Shen P, Zhang D, Luo Y et al. Characteristics of patients with temporomandibular joint idiopathic condylar resorption. Cranio. 2022 Jul 26:1–7. 10.1080/08869634.2022.2100973. Epub ahead of print. PMID: 35880737. 10.1080/08869634.2022.2100973 35880737

[17] Ahmad M, Hollender L, Anderson Q (2009). Research diagnostic criteria for temporomandibular disorders (RDC/TMD): development of image analysis criteria and examiner reliability for image analysis. Oral Surg Oral Med Oral Pathol Oral Radiol Endod.

[18] Wolford LM, Gonçalves JR (2015). Condylar resorption of the temporomandibular joint: how do we treat it? Oral Maxillofac. Surg Clin N Am.

[19] Orzeszek S, Waliszewska-Prosol M, Ettlin D (2023). Efficiency of occlusal splint therapy on orofacial muscle pain reduction: a systematic review. BMC Oral Health.

[20] Chen H, Bi R, Hu Z (2021). Comparison of three different types of splints and templates for maxilla repositioning in bimaxillary orthognathic surgery: a randomized controlled trial. Int J Oral Maxillofac Surg.

[21] Clark GT. The TMJ repositioning appliance: a technique for construction, insertion, and adjustment. Cranio. 1986;4(1):37–46. 10.1080/08869634.1986.11678129 3456393

[22] Ma Z, Xie Q, Yang C, Zhang S (2019). Can anterior repositioning splint effectively treat temporomandibular joint disc displacement?. Sci Rep.

[23] Pihut M, Gorecka M, Ceranowicz P (2018). The efficiency of Anterior Repositioning splints in the management of Pain related to Temporomandibular Joint Disc Displacement with reduction. Pain Res Manag.

[24] Chen X, Li X, Xu L (2016). Development of a computer-aided design software for dental splint in orthognathic surgery. Sci Rep.

[25] Somogyi A, Végh D, Róth I (2023). Therapy for Temporomandibular disorders: 3D-Printed splints from planning to evaluation. Dent J (Basel).

[26] Hua J, Fan X, Nie X (2023). Preliminary evaluation of Kovacs digital occlusal splint in the treatment of temporomandibular disorders: a single-centre, cross-sectional study. J Oral Rehabil.

[27] Farrar WB. Diagnosis and treatment of anterior dislocation of the articular disc. New York J Dentistry 1971 41: 348–51. 5288441

[28] Ha- Na P, Kyoung AK, Kwang- Joon K (2014). Relationship between pain and effusion on magnetic resonance imaging in temporomandibular disorder patients [J]. Imaging Sci Dentistry.

[29] Sato S, Sakamoto M, Kawamura H, Motegi K (1999). Long-term changes in clinical signs and symptoms and disc position and morphology in patients with nonreducing disc displacement in the temporomandibular joint. J Oral Maxillofac Surg.

[30] Roh H-S, Kim W, Kim Y-K, Lee J-Y (2012). Relationships between disk displacement, joint effusion, and degenerative changes of the TMJ in TMD patients based on MRI findings. J Cranio-Maxillofacial Surg.

[31] Dias IM, Cordeiro PC, deF., Devito KL (2016). Evaluation of temporomandibular joint disc displacement as a risk factor for osteoarthrosis. Int J Oral Maxillofac Surg.

[32] Wahaj A, Hafeez K, Zafar MS (2016). Association of bone marrow edema with temporomandibular joint (TMJ) osteoarthritis and internal derangements. CRANIO®.

[33] Eberhard D, Bantleon HP, Steger W. The efficacy of anterior repositioning splint therapy studied by magnetic resonance imaging. Eur J Orthod 2012,24(4):343–52. 10.1093/ejo/24.4.343 12198864

[34] Lei J, Yap AU, Liu MQ (2019). Condylar repair and regeneration in adolescents/young adults with earlystage degenerative temporomandibular joint disease: a randomised controlled study. J Oral Rehabil.

[35] Liu MQ, Lei J, Han JH (2017). Metrical analysis of disc-condyle relation with different splint treatment positions in patients with TMJ disc displacement. J Appl Oral Sci.

[36] Schmitter M, Zahran M, Duc JM (2005). Conservative therapy in patients with anterior disc displacement without reduction using 2 common splints: a randomized clinical trial. J Oral Maxillofac Surg.

[37] Chen HM, Liu MQ, Yap AU (2017). Physiological effects of anterior repositioning splint on temporomandibular joint disc displacement: a quantitative analysis. J Oral Rehabil.

[38] Paradowska-Stolarz A, Wezgowiec J, Malysa A (2023). Effects of Polishing and Artificial Aging on Mechanical properties of Dental LT Clear® Resin. J Funct Biomater.

[39] Paradowska-Stolarz AM, Wieckiewicz M, Mikulewicz M et al. Comparison of the tensile modulus of three 3D-printable materials used in dentistry. Dent Med Probl 2023 Jul-Sep;60(3):505–11. 10.17219/dmp/166070. 10.17219/dmp/166070 37227002

[40] Benli M, Eker Gümüş B, Kahraman Y (2020). Surface roughness and wear behavior of occlusal splint materials made of contemporary and high-performance polymers. Odontology.

[41] Shopova D, Bozhkova T, Yordanova S, Yordanova M, Case, Report (2021). Digital analysis of occlusion with T-Scan Novus in occlusal splint treatment for a patient with bruxism. F1000Res.

